# Supplementary material for: The effects of shoe structural features on agility and stability tasks during walking
Source: PeerJ. 2025 Sep 11;13:e19930. doi: 10.7717/peerj.19930 (PMC12433620; doi:10.7717/peerj.19930)
Supplement: Supplemental Information 1 — We measured participants’ perceptions of performance during each task in each footwear condition using this custom questionnaire. [file peerj-13-19930-s001.pdf]

Participant Puts Shoes On (similar to a shoe store we have them walk a lap around our room and this is to determine if they need any extra support or inserts)

- How do the shoes currently feel for size and arch support? (We then discuss the options for inserts to help improve their fit)
- Do the shoes feel true to your expected size?

Participant Completes the Figure 8's

- Can you share your thoughts with us on how you felt in these shoes during the Figure 8's.
- Is there anything that stood out to you about these shoes whether positive or negative?
- How did the support feel during this task?
- Did you feel like the shoes were comfortable?
- How did these shoes compare to the other ones that you have worn during the collection today?

Participant Completes Walking Trials

- Now that you are walking in the shoes at different speeds, are there any new thoughts that have come up on these shoes from the first task?
- Have any of your previous feelings on these shoes differed?
- Could you discuss with us how the support of the shoe felt during the walking?
- How was the comfort on these shoes during the walking trial?
- How do the current pairs of shoes compare to the others that we have worn?

Participant is Switching Out Shoes for Next Pair (Let the participant hold on look at the shoe while they are answering this next part because some of them like to point to specific things)

- As we switch out shoes, are there any last comments or thoughts that you have about the shoes that you were wearing?
