# Supplementary material for: The effects of shoe structural features on agility and stability tasks during walking
Source: PeerJ. 2025 Sep 11;13:e19930. doi: 10.7717/peerj.19930 (PMC12433620; doi:10.7717/peerj.19930)
Supplement: Supplemental Information 2 — Our primary hypotheses—centered on the overall agility and stability metrics in the full cohort—were pre-specified and powered a priori to detect large effects (Cohen’s d ≈0.80 at 80% power, α = 0.05). The subgroup effect sizes shown are strictly exploratory and were not intended for inferential statistics; they serve to illustrate directionality across age strata rather than to redefine our main findings. [file peerj-13-19930-s002.pdf]

A priori power analysis indicated that a sample size of *n* = 14 would provide 90% power to detect a minimum effect size (Cohen's *d*) of 0.81 at an alpha level of 0.05 in a within-subjects design. Following exclusions due to marker occlusions, the final sample size (*n* = 12) retained sufficient power to detect a slightly larger minimum effect size, with the threshold increasing to Cohen's *d* = 0.89.

In addition, we conducted an exploratory subgroup analysis to compare the directionality of mean differences and effect sizes reported for our primary cohort to those discovered for adults aged 24 to 50, and aged 51 to 75, thereby exploring the potential modifying influence of age.

Effect Sizes for Agility metrics:

	All participants	Ages 24 to 50	Ages 51 to 75
**Step count**	-0.084	-0.392	0.038
**Speed**	0.114	0.072	0.150
**Width of figure 8**	-0.030	-0.165	0.006
**Length of figure 8**	-0.067	-0.154	-0.030

Effect sizes for Stability metrics:

	All participants	Ages 24 to 50	Ages 51 to 75
**Step width**	-0.115	-0.184	-0.120
**Step length**	0.256	1.313	-0.073
**AP MoS**	0.115	1.298	-0.068
**ML MoS**	-0.195	-0.181	-0.233

Our primary hypotheses, which focused on agility and stability metrics across the full cohort, were pre-specified and powered *a priori* to detect large effects (Cohen's d ≈ 0.80 at 80% power, $\alpha = 0.05$). The subgroup effect sizes presented above are exploratory in nature and were not designed to support inferential statistical testing. Rather, they are intended to illustrate potential directional trends across age strata without altering the interpretation of our primary findings. The effect estimates for our pre-registered primary outcomes in the full sample (e.g., average speed: d = 0.114; step length: d = 0.256) remain the central focus of the evidence presented. Although certain subgroup metrics (e.g., figure-8 width, mediolateral margin of stability) yielded smaller or inconsistent effects that fall below our minimum detectable threshold, these findings do not contradict the conclusions drawn from our adequately powered primary analyses and should be interpreted cautiously.
