# Supplementary figures and images for: The effects of shoe structural features on agility and stability tasks during walking
Source: PeerJ. 2025 Sep 11;13:e19930. doi: 10.7717/peerj.19930 (PMC12433620; doi:10.7717/peerj.19930)

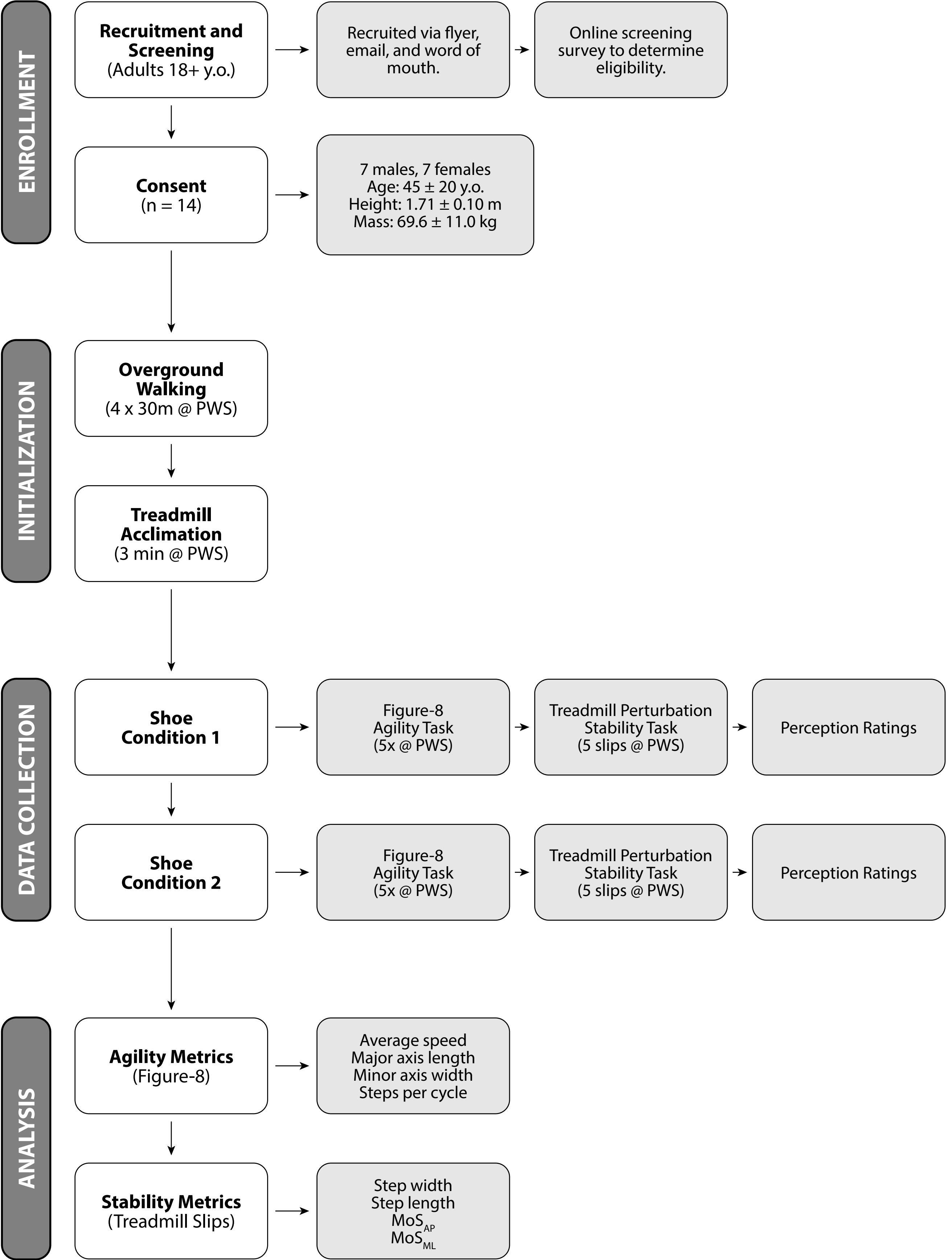

Supplement: Supplemental Information 4 — Schematic diagram of the the experimental protocol and data collection flow. Participants completed treadmill acclimation and overground walking before performing two tasks under each shoe condition: a stability task and an agility task. Outcome measures included step length, step width, margins of stability (MoSAP, MoSML), speed, steps per cycle, and spatial gait metrics. Perception ratings were collected following each condition. [file peerj-13-19930-s004.pdf]
